# Converting tabular data into images for deep learning with convolutional neural networks

**DOI:** 10.1038/s41598-021-90923-y

**Published:** 2021-05-31

**Authors:** Yitan Zhu, Thomas Brettin, Fangfang Xia, Alexander Partin, Maulik Shukla, Hyunseung Yoo, Yvonne A. Evrard, James H. Doroshow, Rick L. Stevens

**Affiliations:** 1 Computing, Environment and Life Sciences, Argonne National Laboratory, Lemont, IL 60439 USA; 2 Frederick National Laboratory for Cancer Research, Leidos Biomedical Research, Inc., Frederick, MD 21702 USA; 3 Developmental Therapeutics Branch, National Cancer Institute, Bethesda, MD 20892 USA; 4 Department of Computer Science, The University of Chicago, Chicago, IL 60637 USA

**Keywords:** Computational models, Machine learning, Virtual drug screening

## Abstract

Convolutional neural networks (CNNs) have been successfully used in many applications where important information about data is embedded in the order of features, such as speech and imaging. However, most tabular data do not assume a spatial relationship between features, and thus are unsuitable for modeling using CNNs. To meet this challenge, we develop a novel algorithm, image generator for tabular data (IGTD), to transform tabular data into images by assigning features to pixel positions so that similar features are close to each other in the image. The algorithm searches for an optimized assignment by minimizing the difference between the ranking of distances between features and the ranking of distances between their assigned pixels in the image. We apply IGTD to transform gene expression profiles of cancer cell lines (CCLs) and molecular descriptors of drugs into their respective image representations. Compared with existing transformation methods, IGTD generates compact image representations with better preservation of feature neighborhood structure. Evaluated on benchmark drug screening datasets, CNNs trained on IGTD image representations of CCLs and drugs exhibit a better performance of predicting anti-cancer drug response than both CNNs trained on alternative image representations and prediction models trained on the original tabular data.

## Introduction

Convolutional neural networks (CNNs) have been successfully used in numerous applications, such as image and video recognition^1–4^, medical image analysis^5,6^, natural language processing^7^, and speech recognition^8^. CNNs are inspired by visual neuroscience and possess key features that exploit the properties of natural signals, including local connections in receptive field, parameter sharing via convolution kernel, and hierarchical feature abstraction through pooling and multiple layers^9^. These features make CNNs suitable for analyzing data with spatial or temporal dependencies between components^10,11^. A particular example is imaging in which the spatial arrangement of pixels carries crucial information of the image content. When applied on images for object recognition, the bottom layers of CNNs detect low-level local features, such as oriented edges at certain positions. While the information flows through the layers, low-level features combine and form more abstract high-level features to assemble motifs and then parts of objects, until the identification of whole objects.

Although CNNs have been applied for image analysis with great success, non-image data are prevalent in many fields, such as bioinformatics^12–14^, medicine^15,16^, finance, and others, for which CNNs might not be directly applicable to take full advantage of their modeling capacity. For some tabular data, the order of features can be rearranged in a 2-D space to explicitly represent relationships between features, such as feature categories or similarities^17–19^. This motivates the transformation of tabular data into images, from which CNNs can learn and utilize the feature relationships to improve the prediction performance as compared with models trained on tabular data. The transformation converts each sample in the tabular data into an image in which features and their values are represented by pixels and pixel intensities, respectively. A feature is represented by the same pixel (or pixels) in the images of all samples with the pixel intensities vary across images.

To our knowledge, three methods have been developed to transform non-image tabular data into images for predictive modeling using CNNs. Sharma et al. developed DeepInsight^17^ that projects feature vectors onto a 2-D space using t-SNE^20^, which minimizes the Kullback–Leibler divergence between the feature distributions in the 2-D projection space and the original full-dimensional space. Then, on the 2-D projection, the algorithm identifies a rectangle that includes all the projected feature points with a minimum area, which forms the image representation. Bazgir et al. developed REFINED (REpresentation of Features as Images with NEighborhood Dependencies)^18^, which uses the Bayesian multidimensional scaling as a global distortion minimizer to project the features onto a 2-D space and preserves the feature distribution from the original full-dimensional space. The features are then assigned to image pixels according to the projection and a hill climbing algorithm is applied to locally optimize the arrangement of feature positions in the image^18^. Ma and Zhang developed OmicsMapNet^19^ to convert gene expression data of cancer patients into 2-D images for the prediction of tumor grade using CNNs. OmicsMapNet utilizes functional annotations of genes extracted from the Kyoto Encyclopedia of Genes and Genomes to construct images via TreeMap^21^, so that genes with similar molecular functions are closely located in the image.

In this paper, we develop a novel method, Image Generator for Tabular Data (IGTD), to transform tabular data into images for subsequent deep learning analysis using CNNs. The algorithm assigns each feature to a pixel in the image. According to the assignment, an image is generated for each data sample, in which the pixel intensity reflects the value of the corresponding feature in the sample. The algorithm searches for an optimized assignment of features to pixels by minimizing the difference between the ranking of pairwise distances between features and the ranking of pairwise distances between the assigned pixels, where the distances between pixels are calculated based on their coordinates in the image. Minimizing the difference between the two rankings assigns similar features to neighboring pixels and dissimilar features to pixels that are far apart. The optimization is achieved through an iterative process of swapping the pixel assignments of two features. In each iteration, the algorithm identifies the feature that has not been considered for swapping for the longest time, and seeks for a feature swapping for it that best reduces the difference between the two rankings.

Compared with three existing methods for converting tabular data into images, the proposed IGTD approach presents several advantages. Unlike OmicsMapNet that requires domain knowledge about features, IGTD is a general method that can be used in the absence of domain knowledge. Because DeepInsight uses the t-SNE projection as image representation, a significant portion of the image is usually left blank, which is composed of pixels not representing features. On the contrary, IGTD provides compact image representations in which each pixel represents a unique feature. Thus, the DeepInsight images are usually much larger than the IGTD images and potentially require more memory and time to train CNNs in subsequent analysis. Compared with REFINED, IGTD generates image representations that better preserve the feature neighborhood structure. In the IGTD image representation, features close to each other in the image are indeed more similar, as will be shown later in the example applications of transforming gene expression profiles of cancer cell lines (CCLs) and molecular descriptors of drugs into images. Also, we take the prediction of anti-cancer drug response as an example and demonstrate that CNNs trained on IGTD images provide a better prediction performance than both CNNs trained on alternative image representations and prediction models trained on the original tabular data. Moreover, IGTD provides a flexible framework that can be extended to accommodate diversified data and requirements. Various measures can be implemented to calculate feature and pixel distances and to evaluate the difference between rankings. The size and shape of the image representation can also be flexibly chosen.

## IGTD algorithm

**Figure 1:**
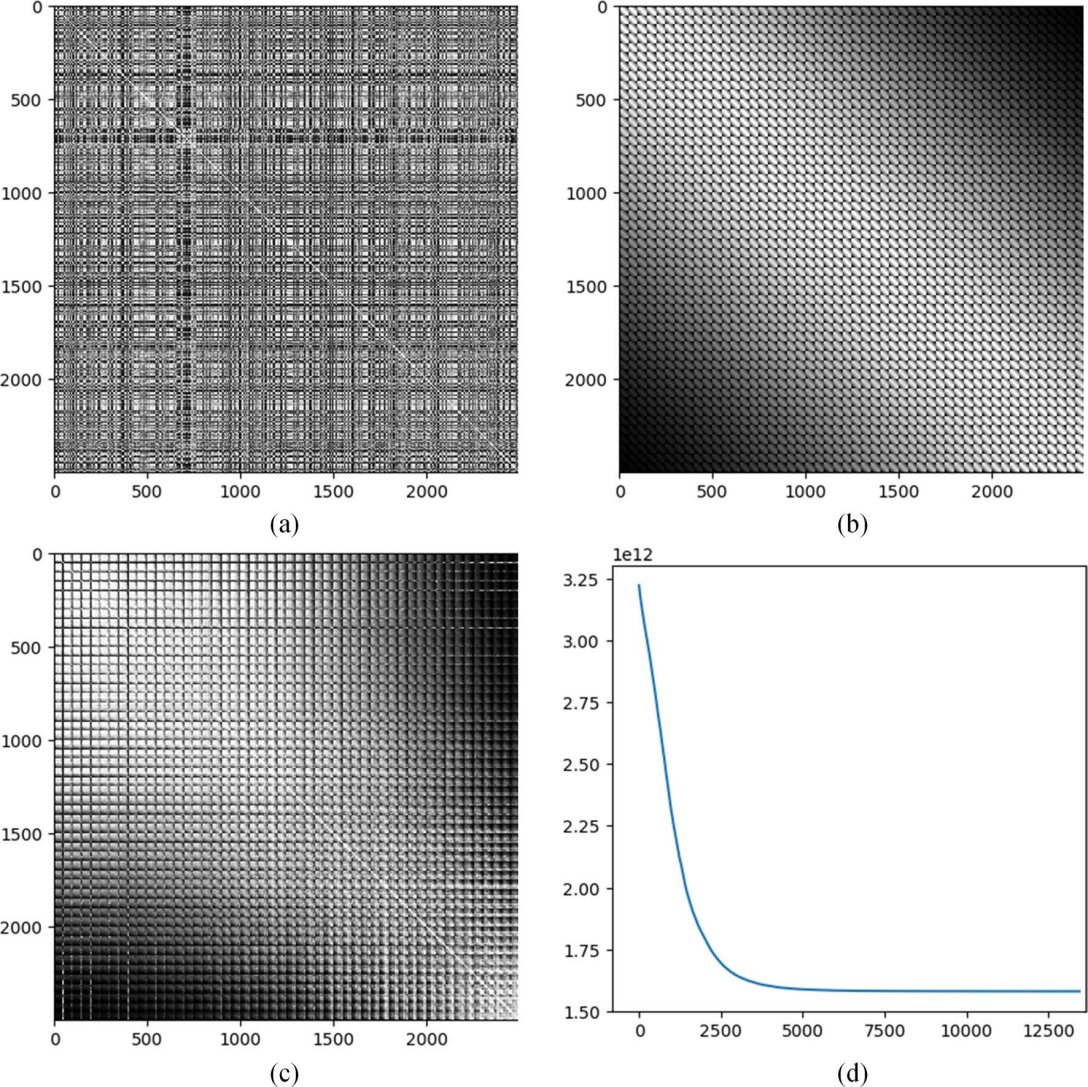
An illustration of IGTD strategy based on CCL gene expression data. (**a**) Rank matrix of Euclidean distances between all pairs of genes. The grey level indicates the rank value. 2500 genes with the largest variations across CCLs are included for calculating the matrix. (**b**) Rank matrix of Euclidean distances between all pairs of pixels calculated based on their coordinates in a $50$ by $50$ image. The pixels are concatenated row by row from the image to form the order of pixels in the matrix. (**c**) Feature distance rank matrix after optimization and rearranging the features accordingly. (**d**) The error change in the optimization process. The horizontal axis shows the number of iterations and the vertical axis shows the error value.

Let ${\varvec{X}}$ denote an $M$ by $N$ tabular data matrix to be transformed into images. Each row of ${\varvec{X}}$ is a sample and each column is a feature. Let ${{\varvec{x}}}_{i,:}$, ${{\varvec{x}}}_{:,j}$, and ${x}_{i,j}$ denote the $i$th row, the $j$th column, and the element in the $i$th row and $j$th column, respectively. The bold upper-case and lower-case letters are used to denote matrices and vectors, respectively. Scalars are denoted by either upper-case or lower-case letters without bold. Our goal is to transform each sample ${{\varvec{x}}}_{i,:}$ into an ${N}_{r}$ by ${N}_{c}$ image (i.e. a 2-D array), where ${N}_{r}\times {N}_{c}=N$. The pairwise distances between features are calculated according to a distance measure, such as the Euclidean distance. These pairwise distances are then ranked ascendingly, so that small distances are given small ranks while large distances are given large ranks. An $N$ by $N$ rank matrix denoted by ${\varvec{R}}$ is formed, in which ${r}_{i,j}$ at the $i$th row and $j$th column of ${\varvec{R}}$ is the rank value of the distance between the $i$th and $j$th features. The diagonal of ${\varvec{R}}$ is set to be zeros. Apparently, ${\varvec{R}}$ is a symmetric matrix. Fig. 1a shows an example of the feature distance rank matrix calculated based on the gene expression profiles of CCLs containing 2500 genes that are taken as features. Details regarding the data will be presented in the next section. Distances between genes are measured by the Euclidean distance based on their expression values. In Fig. 1a, the grey level indicates the rank value. The larger the distance is, the larger the rank is, and the darker the corresponding point is in the plot.

On the other hand, for an ${N}_{r}$ by ${N}_{c}$ image, the distance between each pair of pixels can be calculated based on the pixel coordinates according to a distance measure, such as the Euclidean distance. Then, the pairwise pixel distances are ranked ascendingly. An $N$ by $N$ rank matrix of pixel distances is generated and denoted by ${\varvec{Q}}$, in which ${q}_{i,j}$ is the rank of the distance between pixel $i$ and pixel $j$. The main diagonal of ${\varvec{Q}}$ is set to zeros and ${\varvec{Q}}$ is also a symmetric matrix. The pixels in the image are concatenated row by row to form the order of pixels in ${\varvec{Q}}$. Fig. 1b is an example of the pixel distance rank matrix that shows the ranks of Euclidean distances between all pairs of pixels calculated based on their coordinates in a $50$ by $50$ image. The plot presents two apparent patterns. First, the top right and bottom left corners of the plot are generally darker indicating larger distance and rank values, while the region around the diagonal is generally brighter indicating smaller distances and rank values. Second, the plot shows a mosaic pattern because the pixels are concatenated row by row from the image. Small tiles in the plot correspond to pairwise combinations between rows in the image. Thus, there are totally $50\times 50=\mathrm{2,500}$ tiles in the plot. Each small tile actually shares the same pattern as the whole plot that the top right and bottom left corners of the tile are relatively darker and the region around the diagonal is relatively brighter.

To transform tabular data into images, each feature needs to be assigned to a pixel position in the image. A simple way is to assign the $i$th feature (the $i$th row and column) in the feature distance rank matrix ${\varvec{R}}$ to the $i$th pixel (the $i$th row and column) in the pixel distance rank matrix ${\varvec{Q}}$. But, comparing Fig. 1a with Fig. 1b, we can see the significant difference between the two matrices. An error function is defined to measure the difference

$$\mathrm{err}\left({\varvec{R}},{\varvec{Q}}\right)=\sum_{i=2}^{N}\sum_{j=1}^{i-1}\mathrm{diff}\left({r}_{i,j},{q}_{i,j}\right)$$

where $\mathrm{diff}\left(\cdot ,\cdot \right)$ is a function that measures the difference between ${r}_{i,j}$ and ${q}_{i,j}$, for which there are various options, such as the absolute difference $\left|{r}_{i,j}-{q}_{i,j}\right|$ or the squared difference ${\left({r}_{i,j}-{q}_{i,j}\right)}^{2}$. The error function measures the difference between the lower triangles of the two symmetric matrices. At this stage, the task of assigning each feature to a suitable pixel position so that features similar to each other are close in the image can be converted to reorder the features (rows and columns in ${\varvec{R}}$) so that $\mathrm{err}\left({\varvec{R}},{\varvec{Q}}\right)$ becomes small. Notice that the reordering of rows and columns in ${\varvec{R}}$ needs to synchronized, which means the orders of features along the rows and columns in ${\varvec{R}}$ must always be the same. A basic operation of reordering the features is to swap the positions of two features, because any feature reordering can be implemented by a sequence of feature swaps. Thus, we can reduce the error iteratively by searching for suitable feature swaps. Based on this idea, we design the IGTD algorithm.

The IGTD algorithm takes four input parameters ${S}_{\mathrm{max}}$, ${S}_{\mathrm{con}}$, ${t}_{\mathrm{con}}$, and ${t}_{\mathrm{swap}}$. ${S}_{\mathrm{max}}$ and ${S}_{\mathrm{con}}$ are two positive integers, and ${S}_{\mathrm{max}}\gg {S}_{\mathrm{con}}$. ${S}_{\mathrm{max}}$ is the maximum number of iterations that the algorithm will run if it does not converge. ${S}_{\mathrm{con}}$ is the number of iterations for checking algorithm convergence. ${t}_{\mathrm{con}}$ is a small positive threshold to determine whether the algorithm converges. ${t}_{\mathrm{swap}}$ is a threshold on the error reduction rate to determine whether a feature swap should be performed. The IGTD algorithm takes the following 4 steps.

**Step 1** initializes some variables used in the algorithm. Initialize the iteration index $s=0$. Calculate the initial error ${e}_{0}=\mathrm{err}\left({\varvec{R}},{\varvec{Q}}\right)$. Initialize ${\varvec{h}}$, a vector of negative infinities with a length of $N$. ${\varvec{h}}$ will be used to record the latest iterations in which the features have been considered for feature swap, i.e. in the optimization process ${h}_{n}$ will be the latest iteration in which the $n$ th feature in ${\varvec{R}}$ has been considered for feature swap. Let ${{\varvec{k}}}_{0}$ be $$\left[\begin{array}{ccc}1& \cdots & N\end{array}\right]$$, which indicates the ordering of features at the beginning before optimization.

**Step 2** identifies the feature that has not been considered for feature swap for the longest time and searches for a feature swap for it that results in the largest error reduction. In this step, the iteration index is updated, $s=s+1$. We identify the feature that has not been considered for feature swap for the longest time by identifying the smallest element in ${\varvec{h}}$,

$${n}^{*}=\underset{n\in \left\{\mathrm{1,2},\dots ,N\right\}}{\mathrm{argmin}}{h}_{n}$$

Then we identify the feature whose swap with feature ${n}^{*}$ results in the largest error reduction.

$${l}^{*}=\underset{l\in \left\{1,\dots ,{n}^{*}-1,{n}^{*}+1,\dots ,N\right\}}{\mathrm{argmax}}\mathrm{err}\left({\varvec{R}},{\varvec{Q}}\right)-\mathrm{err}\left({{\varvec{R}}}_{{n}^{*}\sim l},{\varvec{Q}}\right)$$

where ${{\varvec{R}}}_{{n}^{*}\sim l}$ is the matrix resulting from swapping features ${n}^{*}$ and $l$ in ${\varvec{R}}$, i.e. swapping the ${n}^{*}$th and $l$th rows and the ${n}^{*}$th and $l$th columns in ${\varvec{R}}$. In this search, the algorithm repetitively calculates the error reduction resulted from swapping two features. The calculation involves only the rows and columns corresponding to the two features in the feature and pixel distance rank matrices. See Section 1 in the Supplementary Information for more discussion about the calculation.

**Step 3** performs the identified feature swap if the error reduction rate is larger than ${t}_{\mathrm{swap}}$. If $\left(\mathrm{err}\left(R,Q\right)-\mathrm{err}\left({R}_{{n}^{*}\sim {l}^{*}},Q\right)\right)/\mathrm{err}\left(R,Q\right)>{t}_{\mathrm{swap}}$, the algorithm does the following:

(i) ${{\varvec{k}}}_{s}={{\varvec{k}}}_{s-1}$ and swap the ${n}^{*}$ th and ${l}^{*}$ th elements in ${{\varvec{k}}}_{s}$
(ii) $${e}_{s}=\mathrm{err}\left({{\varvec{R}}}_{{n}^{*}\sim {l}^{*}},{\varvec{Q}}\right)$$
(iii) $${h}_{{n}^{*}}=s$$ and $${h}_{{l}^{*}}=s$$
(iv) $${\varvec{R}}={{\varvec{R}}}_{{n}^{*}\sim {l}^{*}}$$

Otherwise, the algorithm does the following:

(xxii) $${h}_{{n}^{*}}=s$$
(xxiii) $${e}_{s}={e}_{s-1}$$
(xxiv) $${{\varvec{k}}}_{s}={{\varvec{k}}}_{s-1}$$

In the case that the identified feature swap is performed, (i) generates the feature reordering indices of iteration $s$ that keep track of feature swap; (ii) calculates the error after feature swap; (iii) labels that features ${n}^{*}$ and ${l}^{*}$ have been considered for feature swap in iteration $s$; (iv) updates the feature distance rank matrix after feature swap. In the case that the feature swap is not performed, (v) labels that feature ${n}^{*}$ has been considered for feature swap in iteration $s$; (vi) keeps the error unchanged from the previous iteration; (vii) keeps the feature reordering indices unchanged from the previous iteration. Notice that if ${t}_{\mathrm{swap}}$ is set to be non-negative, the IGTD algorithm monotonically reduces the error. If ${t}_{\mathrm{swap}}$ is set to be negative, the algorithm has a chance to jump out of a local optimum and search for a potentially better solution.

**Step 4** checks whether the algorithm should terminate or iterate to Step 2 if it should continue. The algorithm runs iteratively and terminates when reaching either the maximum number of interactions ${S}_{\mathrm{max}}$ or convergence where the error reduction rate is continuously smaller than the threshold ${t}_{\mathrm{con}}$ for ${S}_{\mathrm{con}}$ iterations. So, if $s={S}_{\mathrm{max}}$ or $\frac{{e}_{s-{S}_{\mathrm{con}}}-{e}_{u}}{{e}_{s-{S}_{\mathrm{con}}}}<{t}_{\mathrm{con}}$ for $\forall u\in \left\{s-{S}_{\mathrm{con}}+1,\dots ,s\right\}$, the algorithm identifies the iteration with the minimum error

$${v}^{*}=\underset{v\in \left\{1,\dots ,s\right\}}{\mathrm{argmin}}{e}_{v}$$

It then terminates and outputs ${{\varvec{k}}}_{{{\varvec{v}}}^{\boldsymbol{*}}}$ and ${e}_{{v}^{*}}$, which are the optimized indices to reorder the features and the optimized error resulted from reordering the features according to ${{\varvec{k}}}_{{{\varvec{v}}}^{\boldsymbol{*}}}$, respectively. If the termination criteria are not satisfied, the algorithm iterates to Step 2.

### Applications on CCL gene expression profiles and drug molecular descriptors

We applied the IGTD algorithm for anti-cancer drug response prediction. Following existing works^22–24^, we predicted the response of a CCL to a drug treatment using the gene expression profile of CCL and the molecular descriptors of drug. Two benchmark in vitro drug screening datasets, the Cancer Therapeutics Response Portal v2 (CTRP)^25^ and the Genomics of Drug Sensitivity in Cancer (GDSC)^26^, were used to train and evaluate the performance of drug response prediction model. Supplementary Table 1 shows the numbers of CCLs, drugs, and treatments (i.e. pairs of drugs and CCLs) in the two datasets. The IGTD algorithm was used to transform CCL gene expression profiles and drug molecular descriptors into their respective images. A total of 882 CCLs from various cancer types were included in our analysis. Without loss of generality, we chose the 2,500 genes with the largest expression variations across CCLs for analysis. The drugs were represented by chemical descriptors calculated using the Dragon (version 7.0) software package (https://chm.kode-solutions.net/products_dragon.php) based on the drug molecular structure. Molecular descriptors were calculated for a total of 651 drugs included in the two drug screening datasets. Without loss of generality, we also chose the 2500 drug descriptors with the largest variations across drugs for analysis. See Section 2 in the Supplementary Information for the details of data and data preprocessing.

We applied the IGTD algorithm on the CCL gene expression data and the drug molecular descriptors, separately, to generate their image representations. The IGTD algorithm was run with ${N}_{r}=50$, ${N}_{c}=50$, ${S}_{\mathrm{max}}=\mathrm{30,000}$, ${S}_{\mathrm{con}}=500$, ${t}_{\mathrm{con}}=0.000001$, ${t}_{\mathrm{swap}}=0$, the Euclidean distance for calculating pairwise feature distance and pixel distance, and the absolute difference as the $\mathrm{diff}\left(\bullet \right)$ function. Fig. 1a and Fig. 1b show the feature distance rank matrix before optimization and the pixel distance rank matrix, respectively, for the transformation of CCL gene expression profiles into images. Fig. 1c shows the feature distance rank matrix after optimization and rearranging the features/genes accordingly. After optimization the feature distance rank matrix becomes more similar to the pixel distance rank matrix than it originally is. The optimized feature distance rank matrix shares the two important patterns of the pixel distance rank matrix. The top right corner and the bottom left corner in Fig. 1c are relatively dark, while the region around the diagonal is relatively bright, and it also shows a mosaic pattern. The optimization error monotonically decreases and tends to converge after approximately 5,000 iterations as shown in Fig. 1d.

**Figure 2:**
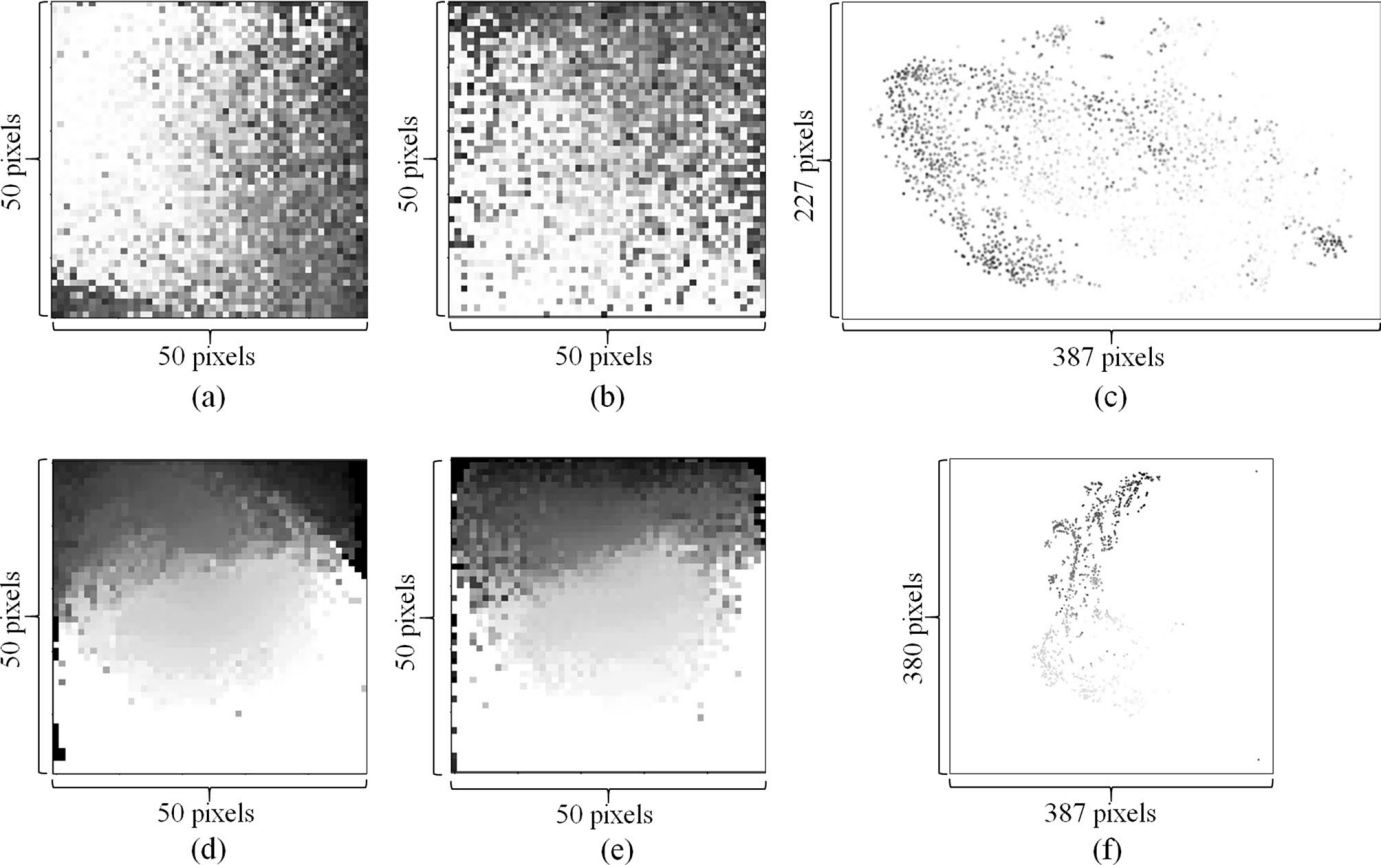
Example image representations of CCL gene expression profiles and drug molecular descriptors. (**a**–**c**) are image representations of the gene expression profile of the SNU-61 cell line generated by IGTD, REFINED, and DeepInsight, respectively. (**d**–**f**) are image representations of molecular descriptors of Nintedanib, generated by IGTD, REFINED, and DeepInsight, respectively.

Based on the optimization results, each gene or drug descriptor was assigned to a pixel in the destination images. The grey level of a pixel in the image indicates the expression value of the corresponding gene in a CCL or the value of the corresponding molecular descriptor in a drug. Fig. 2a shows an example image representation of gene expression profile, which is for the SNU-61 rectal adenocarcinoma cell line (https://web.expasy.org/cellosaurus/CVCL_5078). Fig. 2d shows an example image representation of drug molecular descriptors, which is for Nintedanib (https://en.wikipedia.org/wiki/Nintedanib), an inhibitor of multiple receptor tyrosine kinases and non-receptor tyrosine kinases. In Fig. 2a and Fig. 2d, some genes or drug descriptors have very small values and thus are indicated by white or a color close to white.

For comparison purposes, we also generated image representations using DeepInsight^17^ and REFINED^18^. Fig. 2c and Fig. 2f show the images generated using DeepInsight for the SNU-61 cell line and Nintedanib, respectively. Because the DeepInsight images were generated using 2-D t-SNE projection, a significant portion of the images is blank, especially in the presence of outlier features. To include the 2,500 features into the plots with a reasonable resolution, the size of DeepInsight images are much larger than that of IGTD images, $227\times 387=\mathrm{87,849}$ pixels (Fig. 2c) and $380\times 387=\mathrm{147,060}$ pixels (Fig. 2f) vs. $50\times 50=\mathrm{2,500}$ pixels (Fig. 2a and Fig. 2d). The large images generated by DeepInsight may require more memory and time to train the prediction model in subsequent analysis.

Similar to IGTD, REFINED also generates compact image representations without any blank area. Fig. 2b and Fig. 2e show the images that REFINED generated for the SNU-61 cell line and Nintedanib, respectively. To investigate the difference between IGTD and REFINED images, we used the following local heterogeneity (LH) measure to quantitatively evaluate the preservation of feature neighborhood structure in image representations.

$$\mathrm{LH}=\frac{1}{\left({N}_{r}-p+1\right)\left({N}_{c}-p+1\right)}\sum_{i=\frac{p+1}{2}}^{{N}_{r}-\frac{p-1}{2}}\sum_{j=\frac{p+1}{2}}^{{N}_{c}-\frac{p-1}{2}}\left[\frac{1}{p\times p-1}\sum_{a,b\in {\mathcal{N}}_{i,j}}\left|{y}_{a,b}-{y}_{i,j}\right|\right]$$

where ${y}_{i,j}$ is the intensity of the pixel in the $i$th row and $j$th column of an image (denoted by ${\varvec{Y}}$), and ${\mathcal{N}}_{i,j}$ is a $p\times p$ neighborhood centered around ${y}_{i,j}$ but not including ${y}_{i,j}$. In a $p\times p$ neighborhood, the average absolute difference between the center pixel and the neighbor pixels is calculated to measure the neighborhood heterogeneity. The LH measure is the mean neighborhood heterogeneity obtained by moving the neighborhood window across the whole image. The LH measurements were calculated with multiple neighborhood sizes for both IGTD and REFINED image representations. Two-tail pairwise t-test^27^ was applied across CCLs or drugs to examine the LH difference between IGTD and REFINED images. For each CCL and drug, we also calculated the percentage that IGTD reduced the local heterogeneity compared with REFINED, which is $\left({\mathrm{LH}}_{\mathrm{REFINED}}-{\mathrm{LH}}_{\mathrm{IGTD}}\right)/{\mathrm{LH}}_{\mathrm{REFINED}}\times 100\%$, where ${\mathrm{LH}}_{\mathrm{REFINED}}$ and ${\mathrm{LH}}_{\mathrm{IGTD}}$ are the LH measurements of the REFINED and IGTD images, respectively. Table 1 shows the result. For both CCLs and drugs and all neighborhood sizes in consideration (i.e. 3, 5, 7, and 9), the average LH of the IGTD images is always statistically significantly lower (p-values ≤ 0.05) than that of the REFINED images. This result indicates that the IGTD algorithm better preserves the neighborhood structure of features in the 2-D images, so that similar features are grouped closer in IGTD images.

**Table 1:** IGTD reduces the local heterogeneity (LH) of image representations compared with REFINED.

	Neighborhood size (*p*)	LH (IGTD)	LH (REFINED)	Reduction percentage by IGTD	P-value
CCL	3	0.174 (0.023)	0.187 (0.026)	6.38% (7.08%)	2.05E−107
	5	0.177 (0.024)	0.187 (0.027)	5.32% (6.90%)	3.37E−87
	7	0.179 (0.024)	0.188 (0.027)	4.68% (6.79%)	1.96E−74
	9	0.180 (0.024)	0.189 (0.027)	4.33% (6.57%)	4.65E−69
Drug	3	0.051 (0.013)	0.064 (0.017)	19.99% (4.98%)	1.50E−252
	5	0.056 (0.014)	0.066 (0.017)	14.64% (4.25%)	2.82E−229
	7	0.061 (0.014)	0.069 (0.017)	11.37% (3.92%)	9.87E−199
	9	0.067 (0.015)	0.074 (0.018)	8.63% (3.78%)	4.56E−156

In the LH and reduction percentage columns, the number before the parenthesis is the average value obtained across CCLs or drugs, and the number in the parenthesis is the standard deviation. P-value is obtained via two-tail pairwise t-test comparing the LH between IGTD images and REFINED images across CCLs or drugs.

We also compared the runtimes of IGTD, REFINED, and DeepInsight for converting tabular data into images. For the gene expressions of CCLs, IGTD, REFINED, and DeepInsight took 0.66, 7.69, and 0.04 hour to convert them into images, respectively. For the drug descriptors, IGTD, REFINED, and DeepInsight took 0.74, 5.13, and 0.07 h to convert them into images, respectively. Notice that both IGTD and DeepInsight were executed with one CPU processor, while REFINED was executed with parallel computing using 40 processors of the same specification. This result indicates that DeepInsight converts tabular data into images significantly faster. This observation is expected, because DeepInsight does not generate compact image representations that require an optimization process to assign features to suitable pixel positions as what IGTD and REFINED do. Interestingly, for the two methods that generate compact image representations, the runtimes of REFINED were much longer than those of IGTD, even when REFINED used parallel computing with 40 processors while IGTD used only a single processor.

### Drug response prediction using CNNs based on image representations

**Figure 3:**
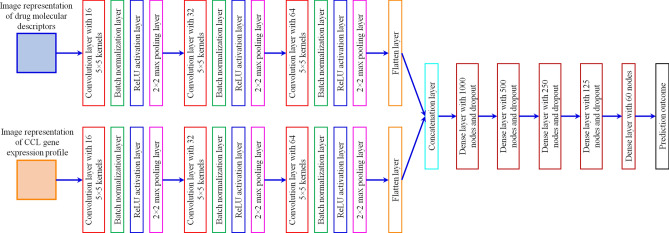
Architecture of the convolutional neural network (CNN) used for predicting drug response based on image representations.

We performed drug response prediction using CNN models trained on the IGTD image representations. See Section 2 in the Supplementary Information for the preprocessing of drug screening datasets. The area under the dose response curve (AUC) was taken as the prediction target in a regression setting. Fig. 3 shows the architecture of the CNN model. For both CCLs and drugs, a subnetwork of three convolution layers, each of which has $5\times 5$ kernels and subsequent batch normalization, ReLU activation, and maximum pooling layers, accepts the image representations as the input. The output feature maps from the subnetworks are flattened, concatenated, and passed to a fully connected network to make predictions. The total number of trainable parameters in the model is 1,307,218. The mean square error was used as the loss function to be minimized during model training. A tenfold cross-validation was performed to train and evaluate the prediction models, in which eight data folds were used for model training, one data fold was used for validation to select the dropout rate and for early stopping to avoid overfitting, and the rest one data fold was used for testing the prediction performance. A total of 20 cross-validation trials were conducted. The prediction performance was measured by the coefficient of determination (R^2^).

To assess the utilities of different image representations, the same CNN models were also trained with REFINED and DeepInsight images. The only difference was when training with DeepInsight images the stride value for moving the convolution kernels was changed from 1 to 2, in order to accommodate the larger input images. Due to the larger input images and consequently larger feature maps and concatenation layer, the number of trainable parameters in the model increased from 1,307,218 for IGTD and REFINED images to 2,715,218 for DeepInsight images. Because the larger input images consumed more memory, we always encountered the out-of-memory error when training models using static data of DeepInsight images. To avoid the error, a data generator mechanism had to be implemented to generate the training data batch by batch on the fly instead of using static data. The out-of-memory error never occurred in model training using static data of IGTD and REFINED images due to their smaller size, which demonstrated that the compact image representations of IGTD and REFINED indeed required less memory for model training.

We also compared CNNs trained on IGTD images with prediction models trained on the original tabular data. Four prediction models, including LightGBM^28^, random forest^29^, single-network DNN (sDNN), and two-subnetwork DNN (tDNN), were included for the comparison. LightGBM is an implementation of the gradient boosting decision tree algorithm that uses techniques of gradient-based one-side sampling and exclusive feature bundling to speed up model training^28^. Random forest constructs multiple decision trees on random sub-samples of data and uses the average of their outcomes as the prediction^29^. sDNN was a fully connected neural network of six hidden layers. For LightGBM, random forest, and sDNN, the CCL gene expression profile and the drug molecular descriptors were concatenated to form the input vector. tDNN was also a neural network with dense hidden layers, but it includes two subnetworks for the input of gene expression profiles and drug molecular descriptors separately. Each subnetwork included three hidden layers. The outputs of the two subnetworks were concatenated and passed to another three hidden layers to make prediction. For a fair comparison, all prediction models were trained and tested through 20 tenfold cross-validation trials, with the same data partitions (i.e. training, validation, and testing sets) used for the cross-validation of CNNs with image representations. See Section 3 in the Supplementary Information for details of the prediction models and the model training process.

Table 2 shows the drug response prediction performance obtained using different data representations and prediction models. CNNs with IGTD images provide the highest average R^2^ across cross-validation trials on both CTRP and GDSC datasets. The average R^2^ of CNN with REFINED images is similar to that of CNN with IGTD images, presumably because both IGTD and REFINED take a similar strategy to generate compact image representations with an intention of grouping similar features together in the image. CNN with DeepInsight images and tDNN with tabular data rank the third and the fourth on the CTRP dataset, while their ranks switch on the GDSC dataset. sDNN, LightGBM, and random forest with tabular data rank the fifth, sixth, and seventh on the two datasets, respectively. The two-tail pair-wise t-test is applied to evaluate the performance difference between CNN with IGTD images and other combinations of prediction models and data representations. The result shows that CNNs trained with IGTD images statistically significantly outperform (p-values ≤ 0.05) all other combinations, except CNNs trained with REFINED images for which the p-values do not make the cutoff.

**﻿Table 2:** Comparison on the drug response prediction performance of different data representations and prediction models.

Dataset	Prediction model	Data representation	R^2^	P-value
CTRP	LightGBM	Tabular data	0.825 (0.003)	8.19E−20
	Random forest		0.786 (0.003)	5.97E−26
	tDNN		0.834 (0.004)	7.90E−18
	sDNN		0.832 (0.005)	1.09E−16
	CNN	IGTD images	**0.856** (0.003)	
		REFINED images	0.855 (0.003)	8.77E−01
		DeepInsight images	0.846 (0.004)	7.02E−10
GDSC	LightGBM	Tabular data	0.718 (0.006)	2.06E−13
	Random forest		0.682 (0.006)	4.53E−19
	tDNN		0.734 (0.009)	1.79E−03
	sDNN		0.723 (0.008)	6.04E−10
	CNN	IGTD images	**0.74** (0.006)	
		REFINED images	0.739 (0.007)	5.93E−01
		DeepInsight images	0.731 (0.008)	2.96E−06

In the R^2^ column, the number before parenthesis is the average R^2^ across 20 cross-validation trials, and the number in the parenthesis is the standard deviation. Bold indicates the highest average R^2^ obtained on each dataset. P-value is obtained via the two-tail pairwise t-test to compare the performance of CNNs trained on IGTD images with those of other combinations of prediction models and data representations.

Because the DeepInsight images are much larger than the IGTD or REFINED images, the number of trainable parameters at least double (2,715,218 parameters vs. 1,307,218 parameters) for CNN models trained on DeepInsight images. To investigate how the larger input image size and consequent model size affect the model training speed, we compare the model training time (i.e. the time to train a prediction model to convergence) of CNNs with different image representations. For each cross-validation trial, we calculate the ratio between the model training time of CNN with DeepInsight or REFINED images and that of CNN with IGTD images. The ratio is then log2 transformed, so that a positive value indicates CNN with DeepInsight or REFINED images takes a longer time to train while a negative value indicates CNN with IGTD images takes a longer time to train. See Table 3 for the mean and standard deviation of the log2 ratio obtained in cross-validation. The one-sample t-test is applied across the cross-validation trials to evaluate how significantly the log2 ratio is different from 0. The result indicates that CNNs take a statistically significantly shorter time (p-values ≤ 0.05) to train on IGTD images than on DeepInsight images for both datasets. CNNs with IGTD images also train statistically significantly faster than CNNs with REFINED images on the GDSC dataset, while their training speeds are similar on the CTRP dataset without a significant difference.

**Table 3:** Comparison on model training time of CNNs trained with different image representations.

Data	Comparison	Log2 ratio of model training time	P-value
CTRP	REFINED vs. IGTD	− 0.034 (0.232)	5.35E−01
	DeepInsight vs. IGTD	4.136 (0.240)	5.55E−25
GDSC	REFINED vs. IGTD	0.172 (0.199)	1.30E−03
	DeepInsight vs. IGTD	4.622 (0.417)	2.34E−21

The number before parenthesis is the average across 20 cross-validation trials, and the number in the parenthesis is the standard deviation. P-value is obtained via the two-tail one-sample t-test across the cross-validation trials.

## Discussion

We developed the Image Generator for Tabular Data (IGTD), a novel algorithm that transforms tabular data into images for deep learning with CNN models. To investigate its utility, we applied the algorithm to convert CCL gene expression profiles and drug molecular descriptors into images, and compared with existing methods that also convert tabular data into images. Compared with DeepInsight, IGTD generates more compact image representations in which every pixel corresponds to a different feature. The compact images reduce the memory consumption and increase the training speed of prediction model in subsequent analysis. As compared with REFINED, the image representations generated by IGTD better preserve the feature neighborhood structure by clustering similar features closer in the images. Based on two benchmark in vitro drug screening datasets, we trained CNNs with the image representations of CCLs and drugs to predict anti-cancer drug response. The prediction performance of CNNs trained on different image representations were compared with each other and with several other prediction models trained on the original tabular data. The results show that CNNs trained on IGTD images provide the highest average prediction performance in cross-validation on both datasets.

IGTD provides a flexible framework that can be easily extended to accommodate diversified data and requirements. Its flexibility can be seen from multiple aspects. First, various distance measures can be designed and used to calculate the feature and pixel distances. For example, besides the Euclidean distance, another feature distance measure is $1-\rho$, where $\rho$ can be a correlation coefficient for continuous variables or the Jaccard index for binary variables. To measure the pixel distance, the Manhattan distance can also be used instead of the Euclidean distance. Second, various difference functions can be implemented to measure the deviation between the feature distance ranking and the pixel distance ranking. Different difference functions may emphasize on distinct aspects of the data. For example, compared with the absolute difference function the squared difference function puts larger weights on elements with large differences. Third, the number of dimensions, size, and shape of the images can be flexibly chosen. The IGTD framework can be extended in a straightforward manner to transform data vectors into not only 2-D matrices, but also 1-D or multi-dimensional arrays with the features rearranged according to mutual similarities or even images of irregular shapes, such as a concave polygon. Fourth, the numbers of features and image pixels can be flexibly adjusted to match each other. If there are more features than image pixels, either larger images with more pixels can be used or a front-end feature selection can be done to reduce the feature number. If there are fewer features than image pixels, either smaller images can be used or pseudo features with all zero elements can be padded to the data to match the feature and pixel numbers.

Compared with existing studies, our IGTD work has the following contributions. First, IGTD transforms tabular data into images using a novel approach, which minimizes the difference between feature distance ranking and pixel distance ranking. The optimization keeps similar features close in the image representation. Second, compared with existing approaches of transforming tabular data into images, IGTD does not require domain knowledge and provides compact image representations with a better preservation of feature neighborhood structure. Third, using drug response prediction as an example, we demonstrate that CNNs trained on IGTD image representations provide a better (or similar) prediction performance than CNNs trained on other image representations and prediction models trained on the original tabular data. Fourth, IGTD is a flexible framework that can be extended to accommodate diversified data and requirements as described above.

Because both IGTD and REFINED generate compact image representations for tabular data, it is important to compare and summarize their difference. We have comprehensively compared the two methods from four aspects, including the local heterogeneity of the generated images, the runtime to generate image representations, the prediction performance based on image representations, and the time for training prediction model. IGTD outperforms REFINED significantly in terms of the preservation of feature neighborhood structure in image and the speed of converting tabular data into images, while the benefit of IGTD is not very significant for improving the prediction performance and the model training speed. Although prediction modeling with CNNs is one of the most important purposes of converting tabular data into images, IGTD also provides a significantly better choice for applications that emphasize on generating compact image representations promptly with a good preservation of feature neighborhood structure.

To understand how sensitive the IGTD algorithm is to the hyper-parameters ${S}_{\mathrm{max}}$, ${S}_{\mathrm{con}}$, and ${t}_{\mathrm{con}}$, we run the IGTD algorithm with three different values for each parameter that spanned across a reasonably large range. Specifically, we tried 10,000, 20,000, and 30,000 for ${S}_{\mathrm{max}}$, 200, 350, and 500 for ${S}_{\mathrm{con}}$, 0.0001, 0.00001, and 0.000001 for ${t}_{\mathrm{con}}$. In total, $3\times 3\times 3=27$ different combinations of parameter settings were used to apply the IGTD algorithm on CCL gene expression profiles and drug molecular descriptors. Supplementary Table 2 shows the optimization results, which are the obtained errors after optimization. To evaluate the variation of error across 27 different parameter settings, we calculated the coefficient of variation for the error, which was the ratio of the standard deviation to the mean. The coefficient of variation of error was 0.029% and 0.039% for the analyses of gene expressions and drug descriptors, respectively. Such small coefficients of variation indicate that the IGTD algorithm is not very sensitive to the variation of the hyper-parameters in a relatively large range. This observation is also expected, because the optimization process reaches a plateau region fairly quickly. For example, in Fig. 1d the error does not change much after about 5000 iterations. As long as the hyper-parameters allow the optimization process to reach the plateau region, the optimization result is not very sensitive to the hyper-parameter setting.

A hypothesis supporting the transformation of data into images is that images may better represent the relationship between features that can be learned by CNNs to facilitate prediction. Apparently, this hypothesis is not universally true for all data. An extreme example can be a dataset including only independent features, where there is no meaningful feature relationship to be represented using images. We expect the IGTD algorithm to perform better for data with feature relationships that can be characterized by feature similarities, although there is not much existing knowledge regarding such relationships.

## Supplementary Information

Supplementary Information.

## Data Availability

IGTD software package is available at https://github.com/zhuyitan/IGTD.
