# Publisher Correction: Converting tabular data into images for deep learning with convolutional neural networks

**DOI:** 10.1038/s41598-021-93376-5

**Published:** 2021-07-01

**Authors:** Yitan Zhu, Thomas Brettin, Fangfang Xia, Alexander Partin, Maulik Shukla, Hyunseung Yoo, Yvonne A. Evrard, James H. Doroshow, Rick L. Stevens

**Affiliations:** 1 Computing, Environment and Life Sciences, Argonne National Laboratory, Lemont, IL 60439 USA; 2 Frederick National Laboratory for Cancer Research, Leidos Biomedical Research, Inc, Frederick, MD 21702 USA; 3 Developmental Therapeutics Branch, National Cancer Institute, Bethesda, MD 20892 USA; 4 Department of Computer Science, The University of Chicago, Chicago, IL 60637 USA

Correction to: *Scientific Reports* https://doi.org/10.1038/s41598-021-90923-y, published online 31 May 2021

The original version of this Article contained an error in the list numerals in the IGTD algorithm section, where,

“Otherwise, the algorithm does the following:

(xxii) $$h_{{n^{*} }}  = s$$
(xxiii) $$e_{s}  = e_{{s - 1}}$$
(xxiv) $$\user2{k}_{s}  = \user2{k}_{{s - 1}}$$”

now reads:

“Otherwise, the algorithm does the following:

(v) $$h_{{n^{*} }}  = s$$
(vi) $$e_{s}  = e_{{s - 1}}$$
(vii) $$\user2{k}_{s}  = \user2{k}_{{s - 1}}$$”

The original Article has been corrected.

