# Supplementary material for: Converting tabular data into images for deep learning with convolutional neural networks
Source: Sci Rep. 2021 May 31;11:11325. doi: 10.1038/s41598-021-90923-y (PMC8166880; doi:10.1038/s41598-021-90923-y)
Supplement: Supplementary file 1 — Supplementary Information. [file 41598_2021_90923_MOESM1_ESM.pdf]

Converting Tabular Data into Images for Deep Learning with Convolutional Neural Networks

(Supplementary Information)

Yitan Zhu[1*], Thomas Brettin[1], Fangfang Xia[1], Alexander Partin[1], Maulik Shukla[1], Hyunseung Yoo[1], Yvonne A. Evrard[2], James H. Doroshow[3], Rick L. Stevens[1,4]

1. Computing, Environment and Life Sciences, Argonne National Laboratory, Lemont, IL 60439, USA

2. Frederick National Laboratory for Cancer Research, Leidos Biomedical Research, Inc. Frederick, MD 21702, USA

3. Developmental Therapeutics Branch, National Cancer Institute, Bethesda, MD 20892, USA

4. Department of Computer Science, The University of Chicago, Chicago, IL 60637, USA

[*]Correspondence: yitan.zhu@anl.gov

Section 1: Calculation of Error Reduction Resulted from Feature Swap

A calculation that the IGTD algorithm repetitively conducts is the error reduction resulted from swapping two features. Supplementary Fig. 1 is an illustration of swapping the positions of two features i and j. The error function is calculated based on the lower triangle of the feature distance rank matrix. Only elements on the orange and blue line segments are affected by feature swap, while all other elements in the lower triangle (blank areas) are not changed. The error reduction can be calculated by the following equation.

$$\begin{aligned}
\mathrm{err}(\boldsymbol{R},\boldsymbol{Q})-\mathrm{err}(\boldsymbol{R}_{i\sim j},\boldsymbol{Q}) = {} & \mathrm{err_v}(\boldsymbol{r}_{i,1:(i-1)},\boldsymbol{q}_{i,1:(i-1)})+\mathrm{err_v}(\boldsymbol{r}_{j,1:(i-1)},\boldsymbol{q}_{j,1:(i-1)}) \\
&+\mathrm{err_v}(\boldsymbol{r}_{j,(i+1):(j-1)},\boldsymbol{q}_{j,(i+1):(j-1)})+\mathrm{err_v}(\boldsymbol{r}_{(i+1):(j-1),i},\boldsymbol{q}_{(i+1):(j-1),i}) \\
&+\mathrm{err_v}(\boldsymbol{r}_{(j+1):N,i},\boldsymbol{q}_{(j+1):N,i})+\mathrm{err_v}(\boldsymbol{r}_{(j+1):N,j},\boldsymbol{q}_{(j+1):N,j}) \\
&-\mathrm{err_v}(\boldsymbol{r}_{j,1:(i-1)},\boldsymbol{q}_{i,1:(i-1)})-\mathrm{err_v}(\boldsymbol{r}_{i,1:(i-1)},\boldsymbol{q}_{j,1:(i-1)}) \\
&-\mathrm{err_v}(\boldsymbol{r}_{i,(i+1):(j-1)},\boldsymbol{q}_{j,(i+1):(j-1)})-\mathrm{err_v}(\boldsymbol{r}_{(i+1):(j-1),j},\boldsymbol{q}_{(i+1):(j-1),i}) \\
&-\mathrm{err_v}(\boldsymbol{r}_{(j+1):N,j},\boldsymbol{q}_{(j+1):N,i})-\mathrm{err_v}(\boldsymbol{r}_{(j+1):N,i},\boldsymbol{q}_{(j+1):N,j})
\end{aligned}$$

where err_v(·,·) is the error function defined for two input vectors of the same length, which is the summation of the differences between the corresponding elements in the input vectors calculated using the given diff(·,·) function.

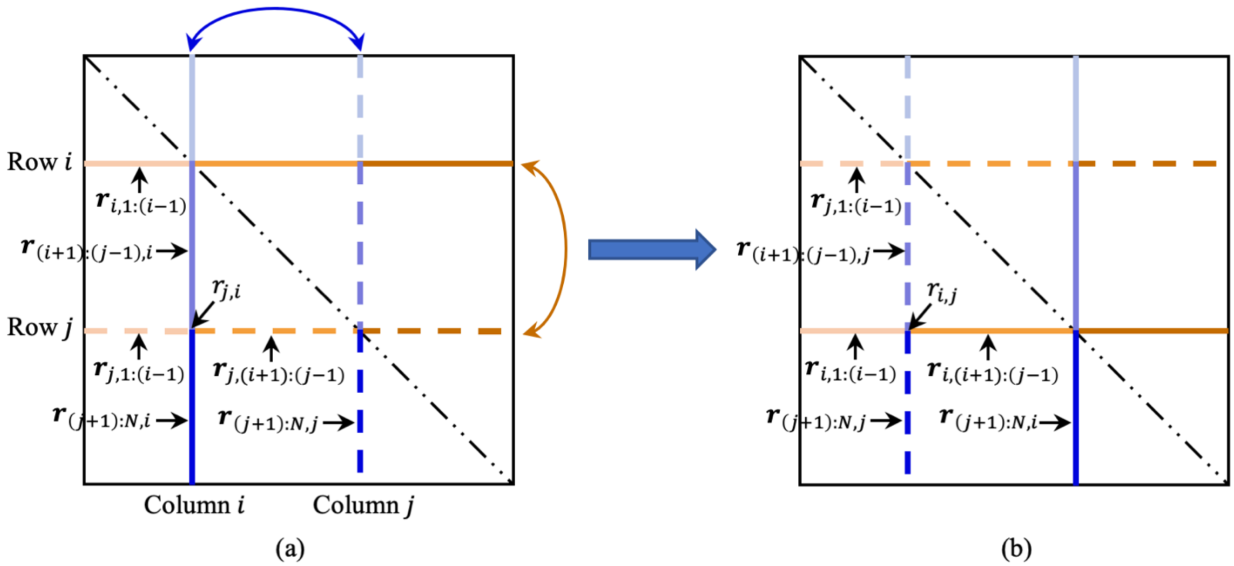

Supplementary Figure 1 Illustration of feature swap on the feature distance rank matrix. Feature i (i.e. row i and column i) and feature j (i.e. row j and column j) are indicated by solid lines and dashed lines, respectively. (a) The situation before feature swap. (b) The situation after feature swap.

Section 2: Details of Data and Data Preprocessing

Drug Screening Data

We applied the IGTD algorithm for anti-cancer drug response prediction. Two benchmark in vitro drug screening datasets, the Cancer Therapeutics Response Portal v2 (CTRP)[1] and the Genomics of Drug Sensitivity in Cancer (GDSC)[2], were used to train the prediction models and to evaluate the prediction performance. Supplementary Table 1 shows the numbers of CCLs, drugs, and treatments (i.e. pairs of drugs and CCLs) in the two datasets. The drug response values in the CTRP and GDSC datasets are measurements of tumor cell viability over multiple doses. We used the three-parameter logistic function (hill slope model) to fit the multi-dose data and generated the dose response curve. The area under the dose response curve (AUC) was calculated for the dose range of [10^{-10}M, 10^{-4}M] as a dose-independent efficacy measure. The AUC value was then normalized by the dose range, so that its value is between 0 and 1, where small values indicate response and large values indicate non-response. Because a pair of drug and CCL may have been tested multiple times in a study, we took an average AUC value for such cases. The numbers of treatments (pairs of drugs and CCLs) in Supplementary Table 1 indicate the numbers of unique combinations of drugs and CCLs in the datasets.

Supplementary Table 1 Numbers of CCLs, drugs, and treatments (pairs of drugs and CCLs) in the datasets.

Dataset	# CCLs	# Drugs	# Treatments
GDSC	659	238	125,712
CTRP	812	494	318,040

Gene Expression Data of Cancer Cell Lines

The gene expression data of CCLs were retrieved from the Cancer Cell Line Encyclopedia (CCLE)[3] online resource, which provides RNA-seq data of all CCLs used in the CTRP and GDSC studies, except 11 GDSC CCLs that were thus excluded from our analysis. Combining the two datasets, a total of 882 CCLs from various cancer types were included in our analysis. Based on the RNA-seq data, TPM (transcripts per kilobase million) values were calculated and a log2 transformation was taken to generate the gene expression values. The resulted transcriptome data included 17,739 genes. Without loss of generality, we chose the 2,500 genes with the largest expression variations across CCLs for analysis. The expression values of each gene were normalized using the follow equations

$$\tilde{x}_{i,j} = \frac{x_{i,j} - v_{\min}}{v_{\max} - v_{\min}}$$

$$v_{\min} = \min_{m \in \{1,\ldots,M\}} x_{m,j}$$

$$v_{\max} = \max_{m \in \{1,\ldots,M\}} x_{m,j}$$

where $x_{i,j}$ is the expression value of the jth gene in the ith CCL and $\tilde{x}_{i,j}$ is its normalized value. After normalization, each gene had a maximum value of 1 and a minimum value of 0.

Drug Molecular Descriptors

The drugs were represented by chemical descriptors calculated using the Dragon (version 7.0) software package (https://chm.kode-solutions.net/products_dragon.php) based on the drug molecular structure. The package calculated various types of descriptors, such as the simplest atom types, functional groups and fragment counts, topological and geometrical descriptors, estimations of molecular properties, and drug-like and lead-like indices. Molecular descriptors were calculated for a total of 651 drugs used in the two drug screening datasets. We kept 3,837 molecular descriptors that did not have missing values. Without loss of generality, we chose the 2,500 descriptors with the largest variations across drugs for analysis. Each drug descriptor was normalized by its minimum and maximum values across drugs in the same way as what was done for normalizing gene expression data.

Section 3: Details of Prediction Models and Model Training Process

CNN Models

We performed drug response prediction using CNN models trained on the image representations of CCL gene expression profiles and drug molecular descriptors. Fig. 4 in the main text shows the structure of the CNN model. For both CCLs and drugs, a subnetwork of three convolution layers, each of which has 5 × 5 kernels and subsequent batch normalization, ReLU activation, and 2 × 2 maximum pooling layers, accepts the image representations as the model input. The output feature maps from the subnetworks are flattened, concatenated, and forwarded to a fully connected network to make predictions. The fully connected network includes five hidden layers with 1,000, 500, 250, 125, and 60 nodes. The hidden layers except the last one has the dropout mechanism for regularization. The dropout rate is the same for all the layers, which is the only hyper-parameter tuned in the model training through cross-validation. The dropout rate was selected from 0, 0.1, 0.25, 0.45, and 0.7 based on the validation loss. The stride used for moving the convolution kernel was always 1, except that it was changed to 2 for training CNNs on DeepInsight images to accommodate the much larger input images. The Adam optimizer with default parameter setting was used for model learning [4]. The learning rate was initialized at 0.001 and was reduced by a factor of 10 if the reduction of validation loss was smaller than 0.000001 in 10 epochs. The training process would stop early if the reduction of

validation loss was smaller than 0.000001 in 20 epochs; otherwise the full training process would take 100 epochs. The analysis pipeline was implemented using the Keras package (https://keras.io/) with Tensorflow (https://www.tensorflow.org/) backend.

Other Prediction Models

We also compared CNNs trained on IGTD images with prediction models trained on the original tabular data. Four prediction models, including LightGBM[5], random forest[6], single-network DNN (sDNN), and two-subnetwork DNN (tDNN), were included for the comparison. For LightGBM, we used the LightGBM python package (https://LightGBM.readthedocs.io/en/latest/index.html) to implement the analysis pipeline. The training of LightGBM model would stop early to avoid overfitting if the validation loss did not reduce in 200 boosting steps; otherwise the whole training process will take 10,000 boosting steps. Default values were used for all other parameters. For random forest, we used the implementation from Scikit-Learn (https://scikit-learn.org/). The prediction model included 1,000 decision trees and default values were used for all other parameters. sDNN was a fully connected neural network of six hidden layers with 2000, 1000, 500, 250, 125, and 60 nodes. tDNN was also a neural network with dense hidden layers, but it included two subnetworks for the input of gene expression profile and drug molecular descriptors separately. Each subnetwork included three hidden layers with 1000, 500, and 250 nodes. The outputs of the two subnetworks were concatenated and forwarded to three hidden layers with 250, 125, and 60 nodes to make prediction. In both sDNN and tDNN models, ReLU was the activation function; all hidden layers except the last one used the dropout mechanism and the dropout rate was the same for the layers. Notice that although sDNN and tDNN had the same total number of nodes in corresponding layers, tDNN had much fewer trainable parameters due to the subnetwork structure. All other parameters and model training mechanism of sDNN and tDNN were the same as those used for the training of CNN models, such as the candidate values of dropout rate, the optimizer and loss function used in model training. For a fair comparison, all prediction models were trained and tested through 20 10-fold cross-validation trials, with the same data partitions (i.e. training, validation, and testing sets) used for the cross-validation of CNNs with image representations.

Section 4: Sensitivity Analysis of Hyper-parameters

We applied the IGTD algorithm on the gene expression profiles of cancer cell lines and molecular descriptors of drugs with different hyper-parameter settings to study its sensitivity to hyper-parameter change. Specifically, we tried 10000, 20000, and 30000 for S_{max}, 200, 350, and 500 for S_{con}, 0.0001, 0.00001, and 0.000001 for t_{con}. In total, $3 \times 3 \times 3 = 27$ different combinations of parameter settings were used to apply the IGTD algorithm. The iterative optimization process of IGTD algorithm aims to minimize an error, which is the difference between the feature and pixel distance rank matrices. Supplementary Table 2 shows the optimization results, which are the obtained errors after optimization. To evaluate the variation of error across 27 different parameter settings, we calculated the coefficient of variation for the error, which was the ratio of the standard deviation to the mean. The coefficient of variation of

error was 0.029% and 0.039% for the analyses of gene expressions and drug descriptors, respectively.

Supplementary Table 2 The obtained errors after optimization for the analyses of gene expressions of cancer cell lines and drug molecular descriptors, when different hyper-parameter settings were used for analysis.

$S_{\max}$	S_{con}	t_{con}	Error after optimization (gene expressions)	Error after optimization (drug descriptors)
10000	200	1.00E-04	1.5825E+12	1.8938E+12
10000	200	1.00E-05	1.5814E+12	1.8925E+12
10000	200	1.00E-06	1.5814E+12	1.8925E+12
10000	350	1.00E-04	1.5818E+12	1.8937E+12
10000	350	1.00E-05	1.5814E+12	1.8925E+12
10000	350	1.00E-06	1.5814E+12	1.8925E+12
10000	500	1.00E-04	1.5817E+12	1.8930E+12
10000	500	1.00E-05	1.5814E+12	1.8925E+12
10000	500	1.00E-06	1.5814E+12	1.8925E+12
20000	200	1.00E-04	1.5825E+12	1.8938E+12
20000	200	1.00E-05	1.5813E+12	1.8923E+12
20000	200	1.00E-06	1.5810E+12	1.8918E+12
20000	350	1.00E-04	1.5818E+12	1.8937E+12
20000	350	1.00E-05	1.5811E+12	1.8921E+12
20000	350	1.00E-06	1.5810E+12	1.8917E+12
20000	500	1.00E-04	1.5817E+12	1.8930E+12
20000	500	1.00E-05	1.5811E+12	1.8920E+12
20000	500	1.00E-06	1.5810E+12	1.8917E+12
30000	200	1.00E-04	1.5825E+12	1.8938E+12
30000	200	1.00E-05	1.5813E+12	1.8923E+12
30000	200	1.00E-06	1.5810E+12	1.8918E+12
30000	350	1.00E-04	1.5818E+12	1.8937E+12
30000	350	1.00E-05	1.5811E+12	1.8921E+12
30000	350	1.00E-06	1.5810E+12	1.8917E+12
30000	500	1.00E-04	1.5817E+12	1.8930E+12
30000	500	1.00E-05	1.5811E+12	1.8920E+12
30000	500	1.00E-06	1.5810E+12	1.8917E+12

References

1 Basu, A. *et al.* An interactive resource to identify cancer genetic and lineage dependencies targeted by small molecules. *Cell* **154**, 1151-1161. https://doi.org/10.1016/j.cell.2013.08.003 (2013).
2 Yang, W. *et al.* Genomics of Drug Sensitivity in Cancer (GDSC): a resource for therapeutic biomarker discovery in cancer cells. *Nucleic Acids Res.* **41**, D955-961. https://doi.org/10.1093/nar/gks1111 (2013).
3 Barretina, J. *et al.* The Cancer Cell Line Encyclopedia enables predictive modelling of anticancer drug sensitivity. *Nature* **483**, 603-607. https://doi.org/10.1038/nature11003 (2012).
4 Ba, J. & Kingma, D. Adam: a method for stochastic optimization. In *International Conference on Learning Representations (ICLR).* (2015).
5 Ke, G. *et al.* LightGBM: a highly efficient gradient boosting decision tree. In *31st International Conference on Neural Information Processing Systems.* 3149-3157 (2017).
6 Breiman, L. Random Forests. *Machine Learning* **45**, 25-32 (2001).
